# Regulating the Vascular Cambium: Do Not Forget the Vascular Ray Initials and Their Derivatives

**DOI:** 10.3390/plants14060971

**Published:** 2025-03-19

**Authors:** Simcha Lev-Yadun

**Affiliations:** Department of Biology & Environment, Faculty of Natural Sciences, University of Haifa—Oranim, Tivon 36006, Israel; levyadun@research.haifa.ac.il

**Keywords:** cambium, differentiation, phloem, rays, stem cells, wood, xylem

## Abstract

The secondary lateral meristem—the vascular cambium (hereafter cambium)—is the largest meristem of the plant kingdom. It is almost always composed of two types of stem cells: (1) the axial (fusiform) initials, the most common and better known and studied, and (2) the ray initials that give rise to the vascular rays (hereafter rays), i.e., the radial component of the secondary xylem and phloem, which are less common and much less studied, and in many studies ignored. There is great flexibility in switching from axial initials to ray initials and vice versa. Ray initials commonly compose ca. 10–40% of the cambium of mature tree trunks, but nothing or very little in typical young model plants used for molecular cambial studies, such as *Arabidopsis thaliana* and young internodes of *Populus* spp. cuttings. I suggest paying more attention to the regulation of the differentiation of ray initials and their derivatives, and to the little-known complicated relations between the axial and ray cambial initials when they contact each other, as well as the special development of pits in their derivatives in cambial molecular studies by using mature trunks of various large woody plants rather than studying *A. thaliana* or young internodes of *Populus* cuttings.

## 1. The Significance of Vascular Land Plants’ Meristems

Several primary and secondary plant meristems (tissues or groups of cells made of stem cells that divide and give rise to other tissues) of vascular land plants directly produce almost all the terrestrial plant biomass and indirectly almost all the terrestrial animal biomass, which directly via herbivory or indirectly via predation by carnivores of herbivores and of smaller carnivores, and by consuming carcasses, feed on the products of these meristems [1,2]. Similarly, except for a minority of about 15% of human nutrition that originates from marine resources (blue food) [3,4], the majority of humanity’s nutrition also originates directly from the meristematic activity of vascular land plants, and indirectly mostly from animals that eat plants, and thus mostly originates in one way or another from the products of land plant meristems. Therefore, studying the biology of these meristems is of enormous importance.

The cambium, a secondary lateral meristem, gives rise to the secondary plant body including the secondary xylem (wood) (see below). The production of secondary wood by the cambium is a critical process in global terrestrial carbon sequestering and in the production of renewable fuel (firewood), a critical essential energy resource for several billion people, while also providing other important industrial resources of plant origin such as timber, and many of the huge fiber masses required for the paper industry [5,6].

While I will focus on the cambial ray initials and their derivatives, because of the global decline of botanical education (see [7]) and the much too frequent botanical confusion I meet as a reviewer and when I read papers, I list below the various vegetative meristems of vascular land plants.

## 2. Primary Meristems

The primary body of the shoot is formed by three primary meristems: (1) the procambium, which gives rise to the primary xylem (both protoxylem and metaxylem) and the primary phloem, (2) the protoderm, which gives rise to the epidermis, and (3) the ground meristem, which gives rise to both the cortex and the pith [8]. In addition to the protoderm and procambium, other variable and flexible primary meristems (probably modifications of the ground meristem) operate in leaves [8,9]. In the primary root, in addition to the above-mentioned three primary meristems, two additional primary meristems are found: the pericycle, which gives rise to lateral roots, and the calyptrogen, which gives rise to the root cap [8]. Alternative names for some of these primary meristems can be found in the literature.

In many species of monocots but also in certain dicots and conifers, an intercalary primary meristem can be found, especially in leaf and internode bases, e.g., [8,10], and it is also found in developing female cone scales of conifers belonging to the Cupressaceae and Taxodiaceae, which have compact, rounded, mature female (seed) cones [11].

## 3. Secondary Meristems

The secondary plant body originates mostly from the cambium, a secondary lateral meristematic tissue, but also from the phellogen (the “cork cambium” that gives rise to the periderm), and from dilatation meristems that produce wedges of additional bark cells, or from dilatation growth by secondary expansion of some of the existing primary cortical cells and many secondary bark cells. The cambium almost always divides inwards to give rise to the wood (secondary xylem), and outwards to give rise to the secondary phloem [8,12], but there are some exceptions to this rule that will not be discussed here. The cambium can also be found and be active to a certain extent in petioles and major leaf veins of leaves of various dicot taxa [12,13].

The cambium should not be confused with the “cork cambium” (phellogen), i.e., the other common but much less active secondary lateral meristem that gives rise to either all or just to most of the cork system in the case of the rhytidome (multilayered cork formed by several phellogens whose cork products repeatedly isolate outer tissues from the inner live tissues and by this eventually cause the death of the outer layers of the primary cortex and of old secondary phloem). The phellogen may originate from many tissues by re-differentiation (commonly referred to in recent years by the problematic term transdifferentiation) of many cell types in various locations within both the primary and secondary plant body [8,14].

The much less studied dilatation secondary meristem operates in the bark and adds wedges of cells in order to avoid bark cracking following the increase in diameter [8,15,16]. Except for anatomical descriptions, i.e., [15], and a basic understanding of their hormonal regulation [16], dilatation meristems are practically terra incognita, waiting for much more discovery of their basic biology and progress.

## 4. Cambial and Ray Structure

The typical cambium of both woody and non-woody plants (including most dicot annuals) typically has two types of initials (stem cells): (1) axial (fusiform) initials, which give rise to the axial components of the secondary plant body (both secondary xylem and secondary phloem), and (2) ray initials, which give rise to the mostly parenchymatic radial component of the secondary xylem and secondary phloem, i.e., the rays [8,12,17,18]. There are certain taxa in which the cambium does not form rays, either only at a young age (temporary raylessness) or ever [17,18]. The formation of ray initials in the cambium is the first, and an easy-to-spot, structural difference between the procambium and the typical cambium [12].

The rays of the secondary xylem are composed, according to taxon and organ age, of one to three types of parenchyma cells: (1) when the cells are axially longer than they are wide, they are termed upright; (2) when they are radially longer than wide, they are termed procumbent; and (3) when they are about as tall as they are wide, they are termed square [19]. The tendency to form more procumbent cells in ray centers is commonly associated with the tree habit [20]. Lev–Yadun and Aloni [17] suggested that, concerning the regulation of the cell orientation of ray parenchyma, upright ray cells represent exposure to a mixture of low radial and high axial developmental signal flow; square ray cells represent approximately equal strength of radial and axial developmental signal flows; and procumbent ray cells represent dominance of a radial developmental signal flow. Herbaceous paedomorphic rays [19] have only or mostly upright ray cells [20,21], since in small herbaceous plants, the cambium is always near the young leaves (strong sources of auxin and other axial developmental signal flows). By contrast, in large woody plants, the cambium is at a substantial distance from these developmental signal sources, and therefore receives lower levels of the axially moving signals. Thus, the radially moving signals become dominant in large woody plants, and therefore their rays are commonly large and have a higher proportion of procumbent cells [17]. Testing this hypothesis requires accurate signal measurements that were technically impossible 30 years ago and are still not easy to carry out, and in addition, many of the developmental signals, including several new hormones known today, were unknown then.

In both conifers and dicots, the longevity of the axial cambial initials depends on their contacts with ray initials ([12,22,23,24], and citations therein). These poorly understood physiological and developmental relationships determine the structure of the cambium and consequently that of its derivatives (the secondary xylem and phloem). The nature of the signals involved in these delicate relationships is unknown and merits discovery.

The axial cells that contact ray cells and the ray cells themselves that contact axial cells have special pitting patterns that differ from those of non-contact cells [19,25,26,27,28]. The regulation of this character, which seems to have various important physiological and ecological functions, is unknown and deserves specific studies.

## 5. Signals That Regulate Cambial Biology

From the fossil record, we know that the cambium, along with its basic hormonal regulation system, which, according to their spiral xylem at branch junctions, included polar auxin transport [29], appeared independently three times during vascular land plant evolution about 400–360 million years ago (the Paleozoic), in three different lineages of seedless vascular plant taxa (lignophytes, lycophytes, and equisetophytes), which previously had only a procambium, producing only primary xylem and phloem [30,31].

In the second half of the 20th century, the differentiation of the cambium itself, its ecologically critical flexible rhythm of activity and dormancy, and the differentiation of the various axial components of the secondary xylem and phloem were mostly attributed to the regulation by the five classic hormones known then: auxin, gibberellin, cytokinin, ABA, and ethylene (e.g., [32,33,34,35,36,37]). The differentiation of the ray initials in the cambium is mostly positively regulated by ethylene [17], although both auxin and gibberellin seem to be involved in the differentiation of certain types of secondary xylem ray cells, such as radial vascular elements (tracheids, vessel members, phloem) and fibers [17,38]. The axial polar auxin flow is involved, however, in regulating ray orientation, shape, splitting, and uniting [17,39].

While the above picture of the regulation of cambial biology that emerged in the 20th century is correct, in recent decades, it has become obvious that it is only basic. With the advent of molecular biology and with the identification of several additional hormones involved in cambial biology, such as brassinosteroids [40], jasmonate [41], strigolactones [42,43], and florigen [44], it has become clear that the basic picture of the regulation of the cambial system by the five classic hormones, which reflects the knowledge of the second half of the 20th century, should be updated. Thus, in recent decades, the involvement of both newly discovered hormones and various non-hormonal regulatory molecules in the differentiation of the cambium from other tissues, its activity, and the differentiation of its derivatives have been studied and considered, e.g., [45,46,47,48]. The current emerging picture is that the regulation of cambial biology is dramatically more complicated. For instance, the most important classic hormone involved in vascular differentiation seems to be auxin, e.g., [32,49]. However, under the title “auxin”, there is a large plethora of transporters such as the *PIN* gene family, e.g., [50,51], and other regulators of its action [48,52,53,54]. Altogether, we are still quite far from a full understanding of cambial biology even in a single model plant, and there is no theoretical reason to dismiss the possibility that different species have various modifications of the basic regulatory system.

## 6. The Cambial Arena

**Figure 1:**
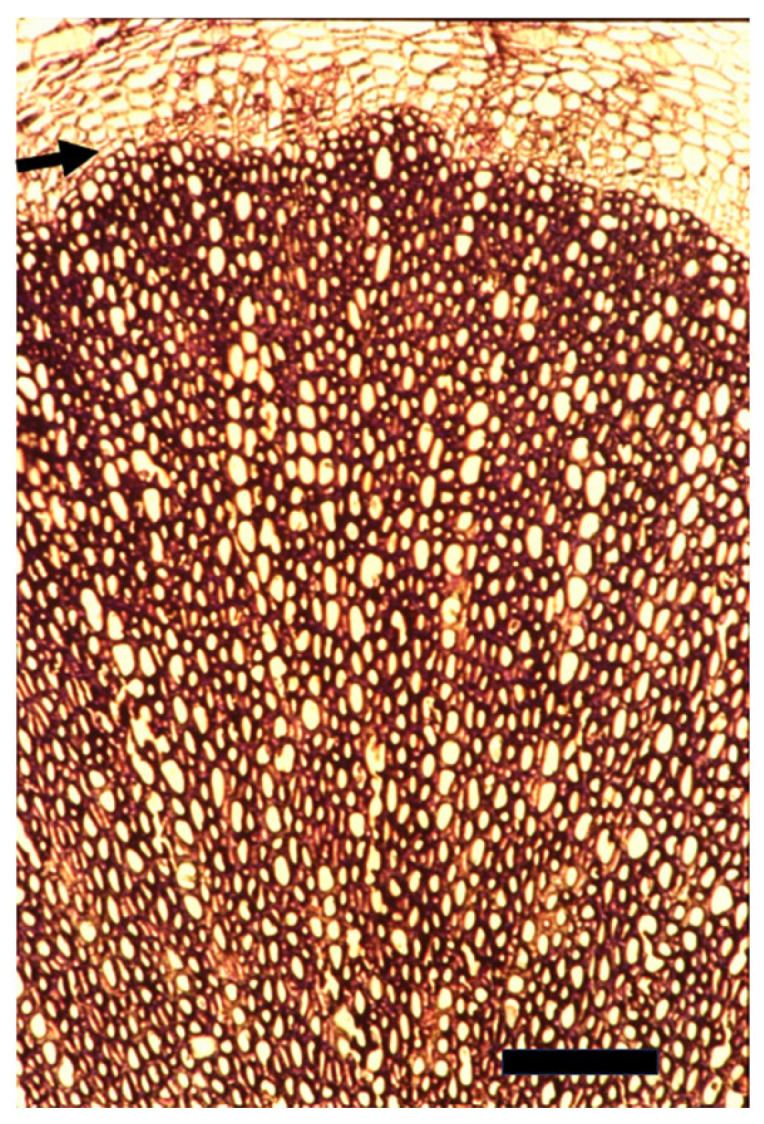
A cross section of a very mature four-month-old main root of a greenhouse-grown large *Arabidopsis thaliana* in which the inflorescent stems were cut off as they appeared in order to delay the monocarpic senescence following flowering. The arrow points to the very thin cambium. The thick secondary xylem is devoid of rays. Bar = 100 μm.

**Figure 2:**
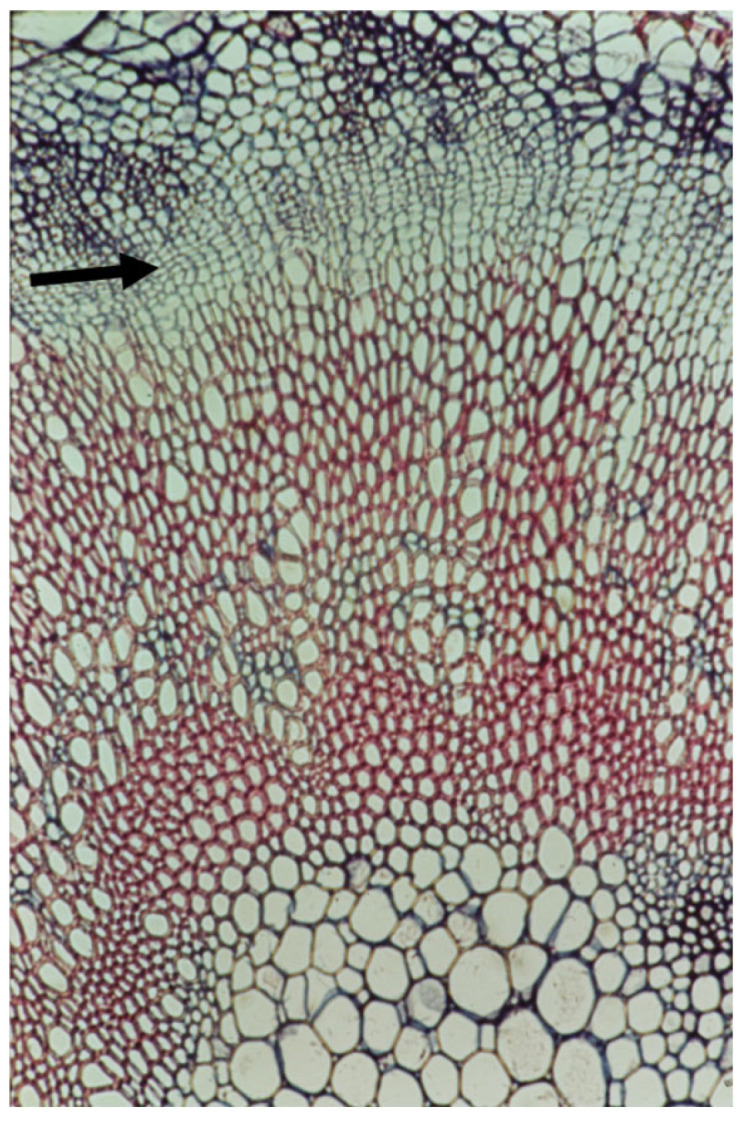
A cross section of the thick bottom of an inflorescence stem of a greenhouse-grown large *Arabidopsis thaliana* in which the inflorescent stems were cut off as they appeared in order to delay the monocarpic senescence following flowering, leaving one inflorescent stem to grow. The arrow points to the multilayered cambium. The thick secondary xylem is devoid of rays.

**Figure 3:**
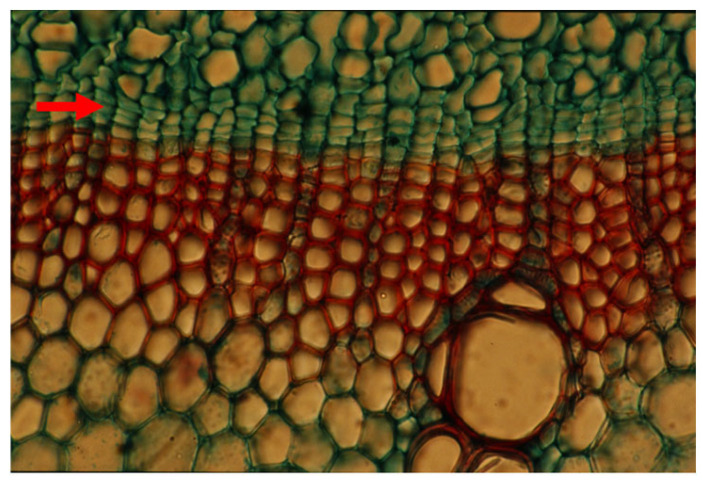
A cross section of a very young stem of *Ricinus communis* showing a multilayered cambium (arrow).

When discussing the cambium from developmental perspectives, there are three arenas/stages: (1) differentiation of the cambium itself from the procambium and interfascicular parenchyma in the shoot, from the procambium and pericycle in the root [8], and from callus following wounding in both shoots and roots [12,55]; (2) the usually flexible rhythm of cambial activity, rest, and dormancy [12,56]; (3) differentiation of the cambial derivatives [8,12]. While each of these arenas/stages may differ from each other in many cases, in many other cases, these stages are a continuum that makes the distinction between them difficult (see [12]). The inherited flexibility of the cambium, as reflected structurally by dramatic changes in the width (in cell file number) of the cambial zone [12], also makes it difficult to distinguish between these three stages in various cases. Therefore, for many decades, there were discussions and disagreements about the number of cambial cell files of initials (stem cells) in the cambium, ranging from a single file of initial cells to several, an issue that will not be discussed here in depth. It is, however, probable that the various and sometimes contrasting results for this important factor of cambial initial cell file number in various studies are related to both the use of specific studied taxa, especially very small and young lab-grown *Arabidopsis thaliana* plants, which typically have a thinner cambium than in trees, and to different seasonal, ecological, and physiological states within individual plants, organs, and cambia. In many tree species, cambial cell divisions occur in several radial cell files and not only in a single one [12] (Figure 1, Figure 2 and Figure 3). Having more than one initial cell file may allow for much better safety in cases of pathogen or herbivore attacks, abiotic stresses, and failures in cell division, and probably also allow for quicker wood production. Theoretically, there is no reason to assume that, concerning the number of cambial initial cell files, all plant taxa, individual plants, and individual cambial sectors within individual plants should be the same.

## 7. The Recent Progress in Understanding Cambial Biology and Overlooking Cambial Ray Initials

Four recent papers [48,57,58,59], like many other recent publications that I do not cite, described the recent progress in studying the cambium, and will be used here to demonstrate some limitations in the use of certain common model plants concerning cambial biology. Kucukoglu et al. [57] used the upper 11- or 18-day-old internodes of cuttings of WT and transgenic hybrid *Populus tremula* × *P. tremuloides* clone T89. Du et al. [58] used several upper very young internodes of two-month-old cuttings of the *Populus alba* × *P. glandulosa* clone 84K. Wybouw et al. [59] reviewed the molecular finds from *Populus* and *Arabidopsis thaliana*, and Eswaran et al. [48] used the main roots of *A. thaliana*. All four above-mentioned studies, like many other non-cited ones, did not perform focused molecular analyses of the rays. The ray initials typically occupy about 10–40% of the cambial area of mature tree trunks [17], and their derivatives compose a similar part of the wood. The rays of the cambium of typical lab-grown small *A. thaliana* plants and young internodes of *Populus* spp. occupy only a minute fraction, if any of the cambium. This results in only a partial understanding of the genes, proteins, and other molecules involved in the typical cambial biology of mature trees and in the differentiation of cambial derivatives of mature trees, and none or only a non-defined portion of the ray-specific ones.

Compared to the typical cambium of mature thick trees used for the paper industry or as a source of timber and firewood (mature pines and many other conifers, poplars, eucalypts, and a large variety of other tropical, subtropical, and temperate dicots), these two common experimental systems either lack the radial (ray) system altogether, or it is just minute and minimal. With all my great appreciation of *A. thaliana* as a model for studying genes involved in wood and fiber formation in trees that I advocated long ago, e.g., [60,61,62], when most relevant scientists thought that *A. thaliana* has no secondary tissues, while using *A. thaliana* has the huge advantage of quickly studying genes cloned from trees because of its short life cycle, small size, and superb genetic knowledge, it cannot represent the many types of wood and the enormous variation in secondary wood structure, physiology, and ecology (see [18,19,63,64]). Unfortunately, *A. thaliana* cannot serve as a model for some of the common and important aspects of wood formation in trees. This limitation includes the absence in the thin *A. thaliana* wood of the large ontogenetic differences in wood structure seen when comparing juvenile wood formed in young and thin trunks, branches, and roots with the wood from the outer layers of mature tree trunks and branches.

## 8. Insufficient Enrichment of Ray Tissue While Sampling for Molecular Studies

Larisch et al. [65] studied the genes involved in the influence of rays on tree and cambial biology in *Populus* × *canescens*. They sampled gene transcripts considered to be enriched for rays by laser microdissection. Their first attempt at a strong enrichment of the ray tissue (Figure 2a in [65]) resulted in degraded RNA. Their second enrichment process of the rays, which provided high-quality RNA, was not focused enough, and their “ray” samples included lots of axial xylem material (see Figure 2 in [65]). In a technically similar paper studying gene expression during cork and xylem formation by laser microdissection of frozen tissues of *Quercus suber* [66], similar problems occurred. The authors showed the sampled tissues in their figure two, and it is clear from that figure that while they enriched the studied target tissues by laser microdissection, the samples still included considerable amounts of other tissues. Accordingly, only wide rays can safely provide samples of clean cambial and ray tissues that will not include non-target axial cambial and vascular cells, something that is currently impossible to achieve with the unicellular rays of *Populus*. Choosing the right model tree other than *Populus* spp., with their too narrow rays, will allow accurate sampling of the ray initials in the cambium, as well as sampling of differentiating and fully differentiated ray cells of the secondary xylem and phloem for gene studies.

The above description of problematic cambial sampling is not the first occurrence of problems of accurate sampling of the cambium for studying regulatory signals. Many dozens of studies from the 1970s until the mid-1990s sampled the cambial zone and obtained a mixture of tissues and cell types, i.e., secondary xylem at several stages of differentiation, cambial initials, and several stages of phloem differentiation (see [67]). A large volume of publications in that issue that I gathered in order to write a review about the hormonal regulation of cambial activity became irrelevant and outdated when Uggla et al. [68] published their much more accurate auxin measurements by using sections of frozen cambial samples that were only 1–2 cells thick.

## 9. Sample Mature Trunks

In a tree 20 m high with a trunk diameter of 50 cm at breast height, the area of the cambium in the trunk, branches, and main roots is in the range of 50 m^2^, a huge arena to experiment on and study when compared to the tiny size of shoot and root apices of *A. thaliana* or of other model plants in which most of the studies on plant stem cells are conducted.

**Figure 4:**
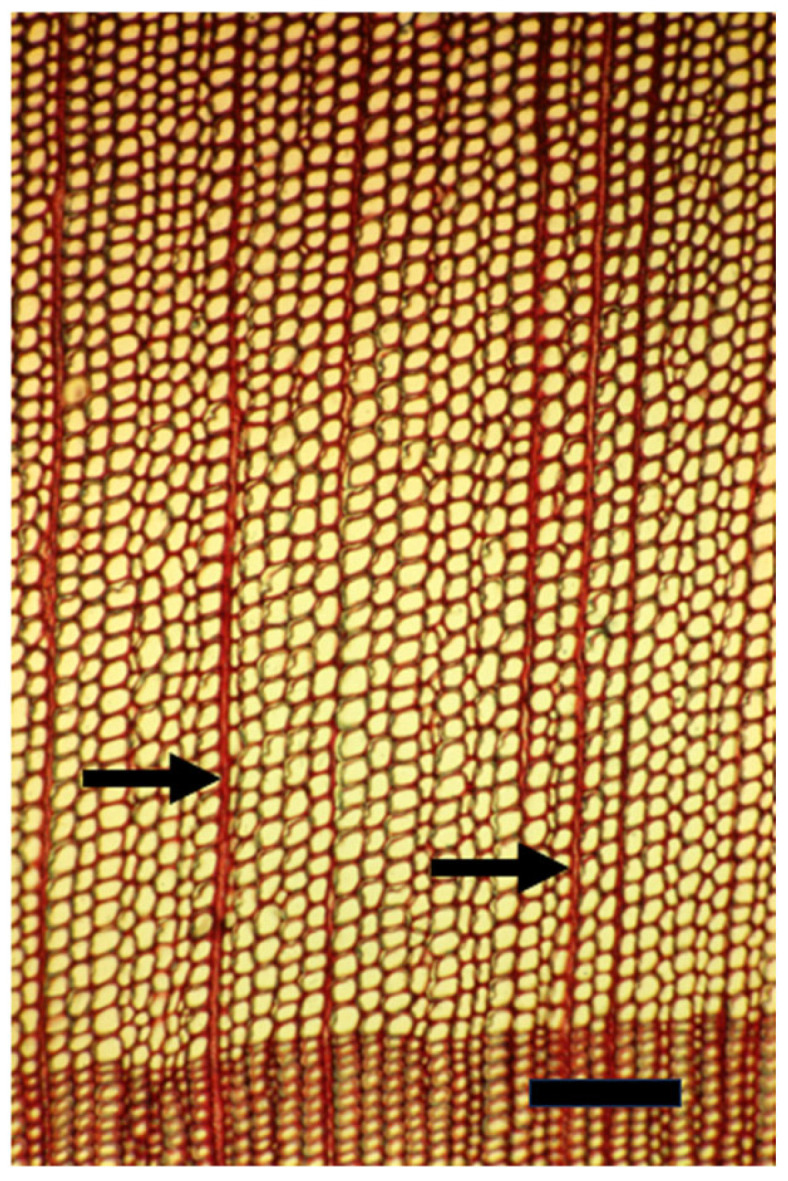
A cross section of a mature trunk of *Pinus halepensis* with its typical thin rays (arrows). Bar = 100 μm.

**Figure 5:**
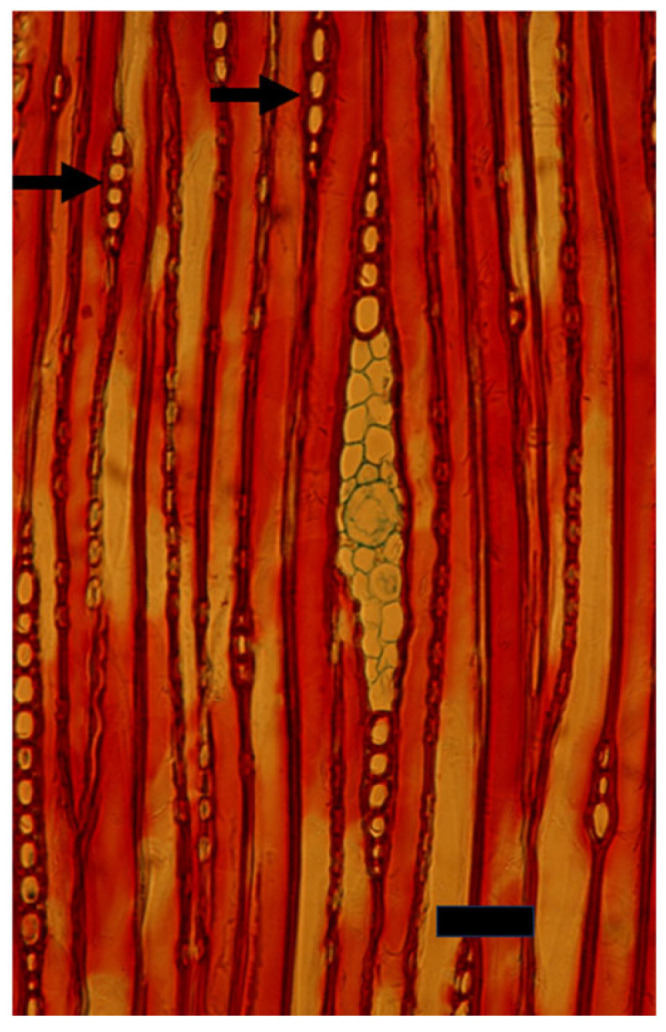
A tangential longitudinal section of a mature trunk of *Pinus halepensis* with its typical thin rays (arrows). A thick ray with a radial resin duct is seen in the center. Bar = 50 μm.

**Figure 6:**
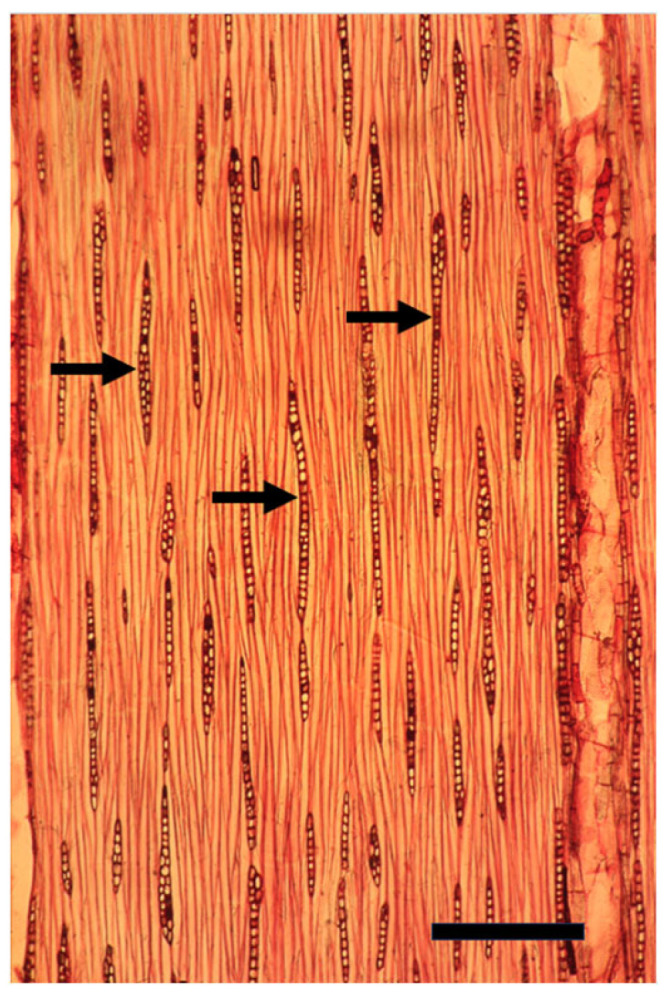
A tangential longitudinal section of the innermost growth ring of a mature trunk of *Melia azedarach* with its typical thin, mostly unicellular rays (arrows). Bar = 200 μm.

**Figure 7:**
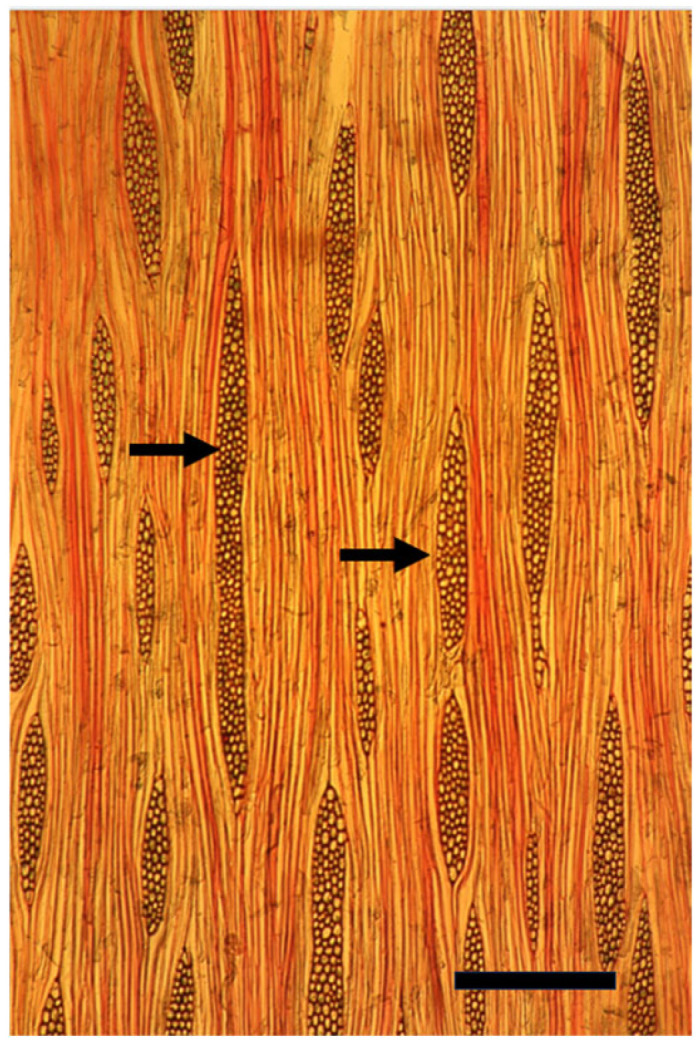
A tangential longitudinal section of a growth ring at 10 cm from the innermost one along the same radius of a mature trunk of *Melia azedarach* with typical thick, multicellular spindle-shaped rays (arrows). Bar = 200 μm.

**Figure 8:**
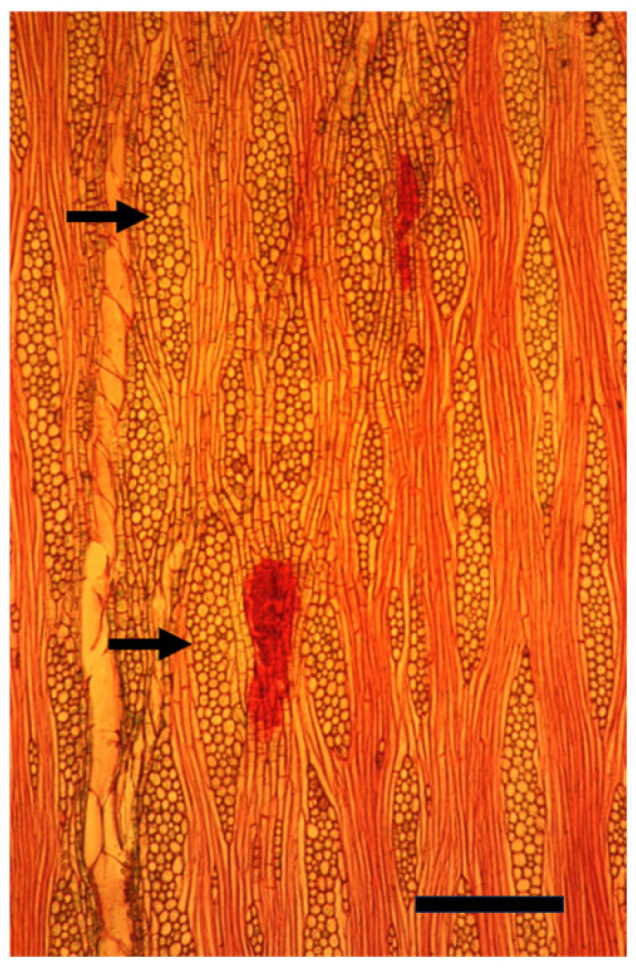
A tangential longitudinal section of a growth ring at 10 cm from the innermost one along the same radius of a mature trunk of *Melia azedarach* treated externally with ethylene with typical much thicker multicellular rays (arrows), with modified shapes, compared to Figure 7. Bar = 200 μm.

**Figure 9:**
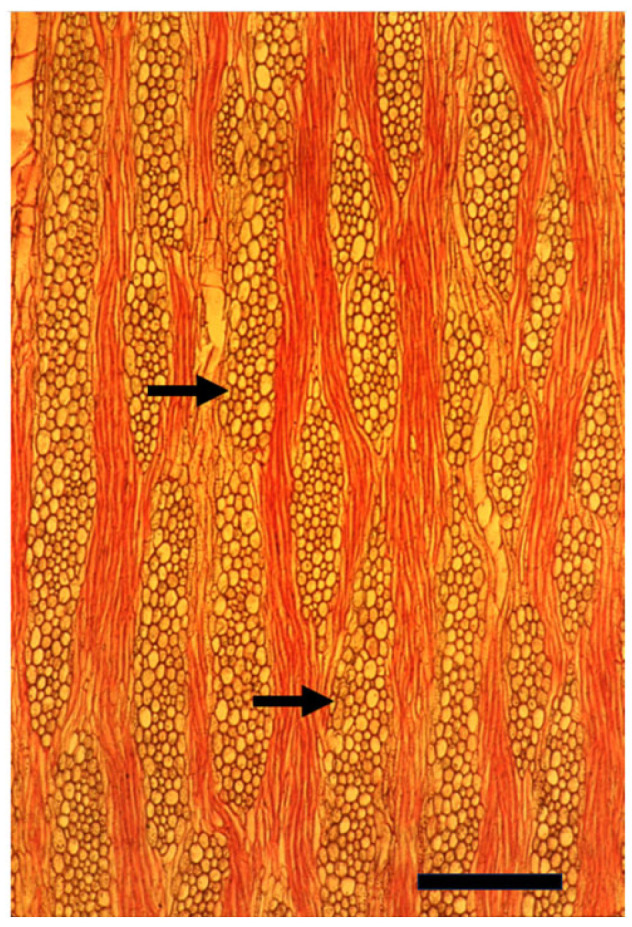
A tangential longitudinal section of a growth ring at 10 cm from the innermost one along the same radius of a mature trunk of *Melia azedarach* wounded with a sharp razor blade with typical much thicker multicellular rays (arrows), with modified shapes, compared to Figure 7, following the involvement of wound ethylene. Bar = 200 μm.

**Figure 10:**
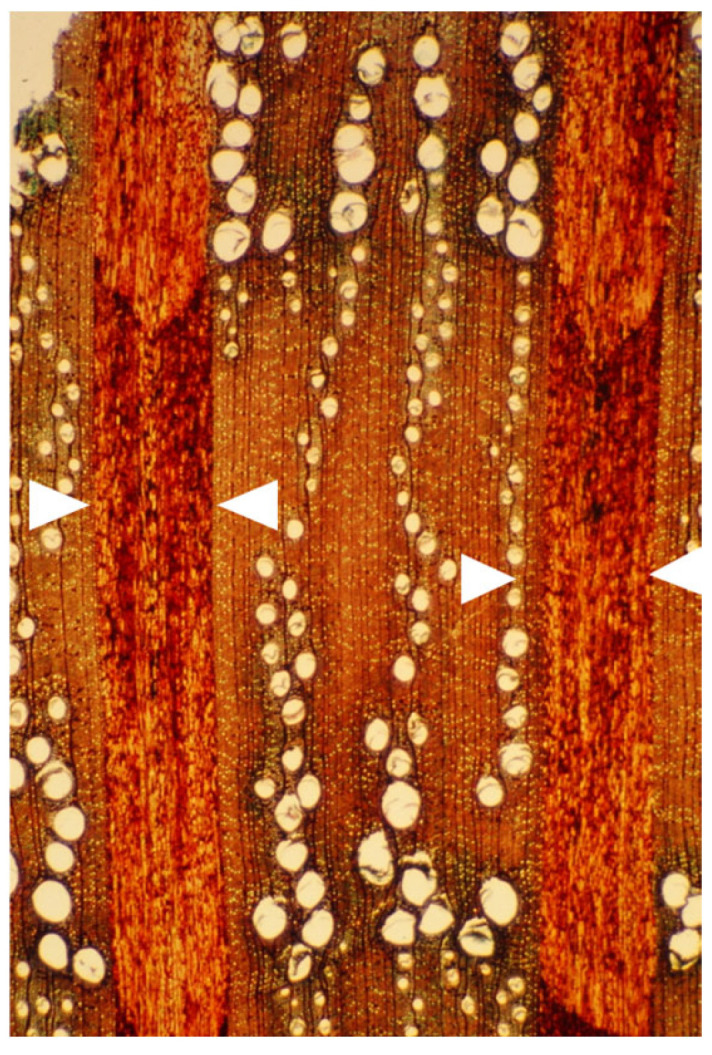
A cross section of a very mature oak (*Quercus ithaburensis*) trunk showing two huge aggregate rays between the arrowheads.

When *Populus* is considered as a model for cambial biology, the common use of very small and young material for molecular studies results in practically missing both the rays and the gradual ontogenetic changes that with the passing years bring about the characters of mature secondary xylem and secondary phloem of *Populus* spp. trunks (see below). Moreover, in the genus *Populus*, like in many conifers, the rays are unicellular, i.e., only one cell wide, making them difficult to sample (Figure 4 and Figure 5). Better models for studying cambial ray initials and their derivatives are the many dicot species that have larger, but non-aggregate rays (see [19,64]) (Figure 6 and Figure 7). It should be remembered that wounding and various experimental procedures induce ethylene production that causes an increase in ray size and number (Figure 8 and Figure 9). Aggregate rays (huge rays formed by the union of smaller rays) of oaks (*Quercus* spp.) and of some other taxa (see [19]) (Figure 10) deserve a separate treatment.

Most current plant scientists are molecular by training, and therefore usually unaware of the phenomenon known as the length-on-age trend of anatomical changes, i.e., the increase in size of tracheids, vessel members, fibers, and rays along the radius with the increasing diameter of a trunk, branch, or root, and in parallel with the increasing distance from the strong developmental signal sources of shoot apices and leaves, e.g., [69,70,71] (Figure 6 and Figure 7). The length-on-age trend must, however, be taken into consideration in many studies of the cambium and its derivatives, not only concerning wood anatomy, ecology, physiology, and evolution, but also in studies of gene expression and activity. When studying the cambium of tree species, in order to avoid studying only juvenile effects, I suggest studying the cambium of trunks that are at least 10–20 cm in diameter, and, whenever possible, even thicker trunks.

In order to get a good picture of the question of the regulation of ray formation, I advocate studying both conifers and dicotyledonous trees, including evergreens, deciduous, temperate, subtropical, and tropical. In order to get a good understanding, the studied taxa should represent all ray types: unicellular, multicellular, aggregate, those with radial resin or gum ducts in the rays and those without such ducts, homocellular and heterocellular rays of various widths, rays of storied cambia, rays of trees with axial alternating fiber and parenchyma bands in the secondary wood such as *Acacia* spp. or *Ficus* spp., taxa with secondary xylem with included phloem, and the cambium of taxa that express temporary raylessness.

## 10. Stem Cells Are Always Differentiated

While many plant scientists believe and even state in writing that plant stem cells including the cambium are not differentiated, in reality, stem cells are always differentiated [72]. For several reasons, the cambium is the easiest and probably the best straightforward model to demonstrate, explain, and study stem cell differentiation: (1) The cambium usually has two stem cell types that differ both structurally and in function (axial and ray initials). (2) In regular shoot ontogeny, the cambium has two different origins from cells that, following their previous differentiation, had different functions (the meristematic procambium and the vegetative interfascicular parenchyma). When the interfascicular parenchyma is considered, these cells have to re-enter the cell cycle when they re-differentiate to a cambium. In the root, however, both origins of the cambium (procambium and pericycle) are meristematic anyway, and the change in their cell biology during their re-differentiation into a cambium is probably smaller. (3) Regenerative cambia in both the shoot and root re-differentiate from a wound callus when they are exposed to an only partly known set of developmental signals.

## 11. Perspectives

In spite of the enormous importance of cambial activity products to global ecology and carbon sequestering, and for providing food and many other important resources for humanity, it seems that much more recent research attention has been given to the easier-to-grow and sample tiny shoot and root growth apices than to the cambium. The genes and other molecules involved in all six cases of forming a cambium from other cell types in the shoot (from procambium and interfascicular parenchyma) and the root (from procambium and pericycle), including regenerative wound cambium from callus in both the shoot and root, or during grafting, are currently only partly known. The same is true for the differentiation of the various specific cell types of the cambial derivatives. Because of the huge ecological and economic value of the cambial derivatives, especially of wood, the cambium in general and its commonly overlooked ray initials and their derivatives deserve much more research attention than they have received, focusing on the use of suitable mature woody model plants.

## Data Availability

In this review no new data were created.
